# Prevalence and renal pathology of pathogenic *Leptospira* spp. in wildlife in Abeokuta, Ogun State, Nigeria

**DOI:** 10.4102/ojvr.v84i1.1210

**Published:** 2017-03-24

**Authors:** Olusola L. Ajayi, Richard E. Antia, Olufemi E. Ojo, Olajoju J. Awoyomi, Latifa A. Oyinlola, Oluwabusola G. Ojebiyi

**Affiliations:** 1 Department of Veterinary Pathology, Federal University of Agriculture, Nigeria; 2 Department of Veterinary Pathology, University of Ibadan, Nigeria; 3 Department of Veterinary Microbiology and Parasitology, Federal University of Agriculture, Nigeria; 4 Department of Veterinary Public health and Reproduction, Federal University of Agriculture, Nigeria; 5 Department of Food Science and Technology, Federal University of Agriculture, Nigeria

## Abstract

There is paucity of information on the prevalence of leptospirosis in wildlife in Nigeria. This study investigated the prevalence and renal pathology of leptospirosis in wild animals in Southwest Nigeria. One hundred and five kidney samples were examined from 10 different wildlife species (antelope) greater cane rat (GCR), hare, African giant rat (AGR), tree hyrax, civet cat, monitor lizard, python, bushbuck and partridge) using a combination of Ellinghausen McCullough Johnson Harris (EMJH) medium, microscopic agglutination test (MAT), Warthin–Starry silver stain (WSss) and immunohistochemistry. Chi-square test was used with confidence level set at 0.05 to ascertain associations between positive cases and sex and species. Eighty-two (78.1%) samples were culturally positive, while 67.7% (63/93), 57.0% (16/28) and 66.7% (8/12) were WSss, MAT and immunohistochemically positive, respectively. Interstitial nephritis (41.0%) and tubular nephrosis (81.0%) were the most prominent histopathological changes. Pathogenic *Leptospira* organisms were highest in GCR (32.1%) and antelope (14.3%). Serovars hardjo (11.54%), bratislava (3.9%), canicola (3.9%), icterohaemorrhagiae (15.4%), pomona (7.14%) gripptotyphosa (19.2%) and undetermined isolates were also detected in other animals. The result showed high prevalence of *Leptospira* infection in the wild and the possibility of domestic animals and humans contracting the disease. This study is the first documentation of evidence of pathogenic *Leptospira* species in wildlife in Nigeria.

## Introduction

Leptospirosis has been adjudged the most common and widespread zoonotic disease in the world (World Health Organization 1999). Over the years, wildlife has been increasingly recognised as the reservoir host and environmental disseminator of different pathogenic leptospires (Chin 2000; Cirone et al. 1978; Cox, Smythe & Leung 2005; Hamir et al. 2001). Increase in disease incidence in domestic animals (especially dogs) and change in serovars involved have been attributed to the endemicity of the disease in wildlife and increase in number of urban wildlife (Okewole & Ayoola 2009; Prescott et al. 2002).

In Nigeria, leptospirosis has been demonstrated serologically in cattle, sheep and goats (Agunloye 2002; Diallo & Dennis 1982; Ezeh et al. 1990) with only one case report of a butcher in which serovar hardjoprajitno was isolated from his urine (Ezeh et al. 1991). In dogs appropriately vaccinated with vaccine containing canicola and icterohaemorrhagiae, serological evidence indicated that there is emergence of new serovars of leptospirosis in Southwest Zone of Nigeria (Okewole & Ayoola 2009).

Despite the zoonotic implication of leptospirosis, little is known of the epidemiology and the health risks of the disease in developing countries, especially in Nigeria where game are regarded as a source of protein. This might be because of lack of awareness and the difficulty associated with the disease recognition and diagnosis.

Current diagnostic methods for leptospirosis in wildlife mostly depend upon demonstration of serum antibodies (Boqvist, Bergstrom & Magnusson 2012; Montagnaro et al. 2010) and a few instances of cultural isolation (CI) (Felt et al. 2011). In the USA, Canada, Trinidad and Tobago, serology, CI and silver impregnation of renal tissues have been used to determine the prevalence of the disease in the wild (Adesiyun et al. 2006; Alton et al. 2009; Everard et al. 1983; Twigg & Cox 1976). The most commonly used serological test is the microscopic agglutination test (MAT). Despite the sensitivity of MAT, it is difficult, labour intensive and sometimes the interpretation of the results is confusing and the test does not indicate active infection (Wild et al. 2002). Immunohistochemistry (IH) has been used in recent times to detect *Leptospira* antigens in the tissues of infected dogs and pinniped populations (Cameron et al. 2008; Ross et al. 2011; Wild et al. 2002). And more recently, IH and polymerase chain reaction were employed as *Leptospira* diagnostic tools in wildlife in Ontario, Canada (Shearer et al. 2014). Presently, studies on the prevalence and renal pathology of leptospirosis in wildlife using the combination of CI, MAT, Warthin–Starry silver stain (WSss) and IH are few in the literature.

Therefore, the paucity of information on leptospirosis in wildlife in Nigeria and the possibility of people contracting the disease through improperly roasted game inspired us to investigate the prevalence of *Leptospira* organism in wildlife, isolate and characterise the prevalent serovars, and examine the renal pathological changes associated with the disease using the combination of CI, MAT, WSss and IH.

## Materials and methods

### Study location

**FIGURE 1:**
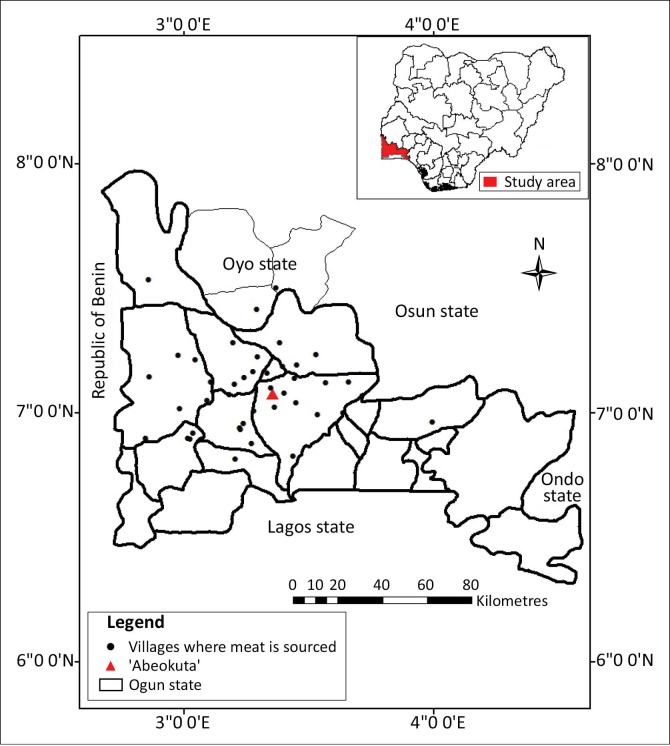
A map of Nigeria showing Abeokuta, Ogun State and the local villages and towns where games are sourced with its neighbouring states and the Republic of Benin.

The study was carried out in Abeokuta (the state capital of Ogun State, Nigeria) and its environs (Figure 1). The city of Abeokuta is located in the southwestern part of Nigeria. Its geographical coordinates are latitude 7° 15’ N and longitude 3° 35‘ E. The average daytime temperature is relatively high, generally above 28 °C, with an annual rainfall of 750 mm and average relative humidity of 74%. The city is about 81 km southwest of Ibadan, the capital of Oyo State, and 106 km north of Lagos, the former capital city of Nigeria. The state shares a boundary on the western part with the Republic of Benin. Abeokuta lies at an altitude of about 157 m a.s.l. (Adekunle & Agbaje 2011).

### Data source

Data were obtained from the wildlife butchers at the Brewery area of Lafenwa, Abeokuta, Ogun State, where hunters from different parts of the state gather to dress and sell their game. The samples were collected between October 2012 and January 2013. People from different part of the state, neighbouring states (especially Lagos, Oyo, Ondo and Osun states) and different parts of the world usually come to buy fresh game for pepper soup, roasted meat (locally called ‘suya’) and smoked meat.

### Sample identification and collection

Ten different species of wildlife were examined and identified by the Department of Wildlife Medicine, Federal University of Agriculture Abeokuta. One hundred and five kidney samples (105) were collected from the 10 different species of wildlife with unknown history, which had been trapped and brought for sale by the wildlife hunters. Twenty-two samples were collected from antelope (*Philantomba walteri*), 54 from greater cane rat (GCR) (*Thyronomys swinderianus*), 11 from hare (*Lepus microtis*), 5 from African giant rat (AGR) (*Cricetomys gambianus*), 3 from tree hyrax (*Dendrohyrax dorsalis*), 3 from civet cat (*Civettictis civetta*), 2 from monitor lizard (*Varanus niloticus*), 3 from python (*Python regius*), and 1 from both bushbuck (*Tragelaphus scriptus*) and partridge (*Perdix perdix*). The sexes of these animals were identified and documented. Visual estimation of their sizes was used to determine their ages, using small, medium and large sizes. The kidneys were collected into ice packs and taken to the Department of Veterinary Pathology, Federal University of Agriculture Abeokuta for subsequent bacteriological and pathological investigations.

## Cultural isolation

### Culture medium

Ellinghausen McCullough Johnson Harris (EMJH) medium (Ellinghausen & McCullough 1965; Johnson & Harris 1967) was used for isolation of the *Leptospira* organisms, modified with the addition of 10% filtered rabbit’s serum (0.2 µm filter). Antibiotics such as chloramphenicol (2 mg/400 mL), nalixidic acid (20 mg/400 mL) and neomycin (4 mg/400 mL) were added and enriched with calcium chloride (1 mL) and magnesium chloride (1 mL) as well as the addition of 5-fluorouracil (400 mg/L) to prevent the growth of other bacteria.

### *Leptospira* isolation

For each kidney, the renal capsule was removed and a tissue sample was taken using a sterile scalpel blade. Tissues were macerated in sterile phosphate buffer solutions (PBS, pH 7.4) using a sterile toothed forceps and then allowed to stay for 10 min. About 5 drops of the tissue extract were inoculated into the medium and incubated at room temperature (28 °C – 30 °C) in the dark. The culture medium was examined after 24 hours for the growth of *Leptospira* organism and later weekly under dark field microscopy.

### Characterisation of *Leptospira* isolates using microscopic agglutination test

Twenty-eight uncontaminated isolates were characterised as previously described (Obregón et al. 2007) using six monoclonal antibodies (mAbs) (Table 1). The serogroups of mAbs used include canicola, icterohaemorrhagiae, bratislava, grippotyphosa, pomona and hardjo. A 50% reduction in the number of free leptospires in the test sample was considered positive with or without agglutination and was recorded as the respective titre (Senthil, Ramadass & Nachimutu 2001).

**TABLE 1:** Reference rabbit leptospiral antisera used for characterisation of leptospiral isolates from wildlife.

Serogroup	Serovar	Strain
Canicola	Canicola	Hond Utrecht IV
Icterohaemorrhagiae	Icterohaemorrhagiae	RGA
Pomona	Pomona	Pomona
Grippotyphosa	Grippotyphosa	Moskva V
Sejroe	Hardjo type Prajitno	Hardjoprajitno
Australis	Bratislava	Jez Bratislava

#### Pathological changes

Samples of the kidney were grossly and histopathologically examined. They were collected into neutral buffered 10% formalin and processed via standard paraffin-embedding techniques. Sections of the 105 kidney samples were cut at 5 µm and stained with haematoxylin and eosin stain, while 93 sections were stained with WSss. Gross and histopathological grading of the observed lesions was performed. The severity of the gross lesions was graded as absent (-), mild (+), moderate (++) and marked (+++). For interstitial nephritis (IN), scores were assigned as follows: + = 1–2 foci on section examined, ++ = 3–4 foci on section examined, +++ = > 5 foci on section examined. The severity or density of *Leptospira* organism using WSss was determined according to the method of Twigg and Cox (1976) with slight modification using mild (+) for 1–2 foci with low density of the organism forming a thin layer on the apical part of tubular epithelial cells, moderate (++) for 3–4 foci with denser mass in the form of a thick rope around the lumen with a clear centre and marked (+++) = > 5 foci with entire lumen occluded by a tangled mass of *Leptospira* organisms. The severity of tubular colonisation and zonal localisation (cortex, medulla and corticomedullary junction) of *Leptospira* infection was also determined.

#### Immunohistochemistry

Three samples each were selected from four different species, namely antelope, hare, AGR and GCR for IH. The rabbit immune sera-cocktail (Table 1) used as primary antibodies against *Leptospira* antigens in the kidney sections were graciously provided by Prof. R.A. Hartskeerl (WHO/FAO/OIE and National Leptospirosis Reference Centre, KIT Biomedical Research, Amsterdam, the Netherlands). IH was performed as previously described (Ross et al. 2011). All steps were performed at room temperature. The sections were first incubated in 3% hydrogen peroxide for 15 min to quench endogenous peroxide. After a brief wash of TPBS, Histomark (Biotin streptavidin-HRP System, Goat anti-Rabbit IgG [H+L] KLP, Gaitherburg, USA) detection system was used. Non-specific binding was done by bathing in normal goat serum for 10 min. The tissues were then incubated with specific mAb (1:800 dilutions in PBS) for 30 min. After a last wash in TPBS, the slides were incubated with streptavidin–biotin–horseradish peroxidase for 15 min. Slides were then rinsed with distilled water and incubated with 3-amino-9-ethylcarbazole-peroxidase chromogen for 10 min, rinsed with distilled water and counterstained with Mayer haematoxylin for 90 s, rinsed and mounted with glycerol for microscopic examination.

### Statistical analysis

Descriptive statistics were used for both positive and negative cases for different levels of age, sex and species. Chi-square test of association was used with confidence level set at 0.05 to ascertain associations between positive cases and sex and species of animals involved.

## Results

The prevalence of *Leptospira* organism according to sexes and sizes in the wildlife using EMJH medium is depicted in Table 2. Eighty-two (78.10%) samples were culturally positive for *Leptospira* infection out of the 105 kidney samples examined. Out of the 22 Antelopes, 18 (81.80%) were positive and 4 (18.20%) were negative. Twenty-seven (50.00%) of the 54 GCRs were positive, while 27 (50.00%) were negative. Of the 11 hare samples examined, 7 (63.64%) were positive, while 4 (36.36%) were negative. Out of the 5 and 3 samples examined from AGR and tree hyrax, 4 (80.00%) and 2 (67.00%) were positive, respectively, and only 1 sample was negative in both species. Two and 3 samples were examined from monitor lizard and python, respectively, with 100% positivity in each case. One kidney sample each was examined in the bushbuck and partridge and both were positive.

**TABLE 2:** The prevalence of Leptospira organisms in 105 kidneys of wildlife in Ellinghausen McCullough Johnson Harris medium according to sex and size.

Species	Total	Number +ve	% +ve	Number -ve	% -ve	Sex				Size					
	
						Male		Female		Small		Medium		Large	
				
						+ve/%	-ve/%	+ve/%	-ve/%	+ve/%	-ve/%	+ve/%	-ve/%	+ve/%	-ve/%
Antelope	22	18	81.80	4	18.20	07/17.5	02/16.7	11/26.2	02/18.1	-	-	4.0/12.0	-	13/46.4	05/71.4
Greater cane rat	54	41	50.00	13	50.00	23/57.5	09/75.0	18/42.9	04/36.4	08/38.0	06/66.7	23/69.7	06/85.7	10/35.7	01/14.3
Hare	11	7	63.64	4	36.36	06/15.0	-	01/2.4	04/36.4	06/28.5	02/22.2	02/6.1	01/14.3	-	-
African giant rat	5	4	80.00	1	20.00	01/2.5	01/8.3	03/7.1	-	04/19.1	01/11.1	-	-	-	-
Tree hyrax	3	2	66.67	1	33.33	-	-	02/4.8	01/9.1	01/4.8	-	-	-	01/3.6	01/14.3
Bushbuck	1	1	100.00	-	-	01/2.5	-	-	-	-	-	-	-	01/3.6	-
Monitor lizard	2	2	100.00	-	-	-	-	02/4.8	-	-	-	02/6.1	-	-	-
Partridge	1	1	100.00	-	-	-	-	01/2.4	-	01/4.8	-	-	-	-	-
Python	3	3	100.00	-	-	01/2.5	-	02/4.8	-	-	-	-	-	03/10.7	-
Civet cat	3	3	100.00	-	-	01/2.5	-	02/4.8	-	01/4.8	-	02/6.1	-	-	-

**Total**	**105**	**82**	**78.10**	**23**	**21.90**	**40**	**12**	**42**	**11**	**21**	**9**	**33**	**7**	**28**	**7**

+ve, positive; -ve, negative; -, absent; % -ve, percentage negative; % +ve, percentage positive.

Of the 105 animals, 52 (49.5%) were males and 53 (50.5%) were females. Of the 52 male samples, 40 (76.9%) were positive, while 42 (79.3%) were positive of the 53 female samples. GCR had the highest infection prevalence of 57.5% compared with 17.5% and 15% in antelopes and hare, respectively. Of the 42 positive females, GCR also showed the highest prevalence of 42.9%, while antelope and AGR had 26.2% and 7.1%, respectively. Other animals such as tree hyrax, monitor lizard, python and civet cat had 4.8% prevalence each. The hare and partridge showed the least prevalence of 2.4% each among the positive female animals. There were no significant (*p* > 0.05) sex and species differences in *Leptospira* prevalence.

Of the 105 kidney samples, 30 animals (28.5%) were of small size and 40 (38.1%) medium size, while 35 (33.4%) were of large adult size. Of the juvenile small-sized animals, 21 (70.0%) were positive; GCR and hare had the highest prevalence of 8 (38.0%) and 6 (28.5%), respectively. Among the medium-sized animals, 33 (82.5%) were positive. Antelope and GCR had the highest prevalence of 4 (12.0%) and 23 (69.7%), respectively. Within the 35 large-sized adults, 28 (80.0%) were culturally positive, with antelope and GCR having the highest prevalence of 46.4% and 35.7%, respectively.

Table 3 depicts the characterisation and distribution of the *Leptospira* isolates from different wild animals. Of the 82 cultured isolates, 28 uncontaminated samples were used for MAT. *Leptospira grippotyphosa* had the highest isolates of 5 (19.23%) with agglutination titre of 1:3200. Two of the 5 *L. gripptotyphosa* isolates were isolated from GCR, while 1 each was isolated from python, antelope and AGR. This was closely followed by *Leptospira hardjo* with 4 (15.38%) isolates; 3 (10.70%) from GCR and 1 from antelope with agglutination titre of 1:1600. There were three (10.70%) *Leptospira icterohaemorrhagiae* isolates from 2 GCRs and 1 partridge with agglutination titre of 1:1600. *Leptospira pomona* was 2 (7.00%) isolates, isolated from 2 antelopes with agglutination titre of 1:3200. One each of (4.00%) *Leptospira bratislava* and *Leptospira canicola* was isolated and characterised from two GCRs and showed agglutination titre of 1:1600 and 1:800, respectively.

**TABLE 3:** Prevalence and characterisation of 28 Leptospira isolates of wild animals using monoclonal antibodies in the microscopic agglutination test.

Number	Serovars	Number +ve	Prevalence (%)	Agglutination titre	Antelope (*n* = 6)	Greater Cane rat (*n* = 15)	Hare† (*n* = 2)	African giant rat (*n* = 1)	Bushbuck (*n* = 1)	Partridge (*n* = 1)	Python (*n* = 2)
1	*Leptospira pomona*	2	7.14	1; 3200	2	-	-	-	-	-	-
2	*Leptospira grippotyphosa*	5	19.23	1; 3200	1	2	-	1	-	-	1
3	*Leptospira hardjo*	4	11.54	1; 400	1	3	-	-	-	-	-
4	*Leptospira bratislava*	1	3.85	1; 1600	-	1	-	-	-	-	-
5	*Leptospira canicola*	1	3.85	1; 800	-	1	-	-	-	-	-
6	*Leptospira icterohaemorrhagie*	3	15.38	1;1600	-	2	-	-	-	1	-
	Determined isolates	16	57.10	-	4	9	-	1	-	1	1
	Undetermined isolates	12	42.90	-	2	6	2	-	1	-	1

+ve, positive; -, absent;

† , contamination and undecided.

Of the 105 kidney samples collected, only 24 (22.90%) showed visible gross lesions. The type, severity and the distribution of the lesions are depicted in Table 4. The gross changes revealed 2 kidneys with multiple foci of cortical haemorrhages from 1 antelope and 1 monitor lizard, while 8 (6.86%) animals (2 antelopes, 4 GCRs and 2 AGR) had moderate-to-severe rough and pitted cortical surfaces with adherence of the renal capsule. Mild-to-moderate cortical necrosis in 9 (5.88%) animals (5 antelopes, 2 GCRs and 1 each from AGR and bushbuck) and 2 (1.96%) moderate multiple foci of pale nodules from antelope and GCR were observed. A moderate locally extensive red infarct was observed in the kidney of 1 tree hyrax and 2 renal hypoplasia from 1 GCR and 1 hare. Of the 24 samples with gross lesions, 18 (75.00%) were culturally positive, while 64 (70.00%) from the remaining 81 samples without macroscopic lesions were also positive. Of the 18 culturally positive samples with gross lesions, 7 each were from antelopes and GCRs, while 2 were from AGRs and 1 each from bushbuck and monitor lizard.

**TABLE 4:** Gross morphologic lesions of the kidney in relation to cultural isolation of *Leptospira* organisms.

Species	Total	Number +ve	Gross morphological changes						CP/PL (%)	CP/AL (%)

			CH	PRCS	RN	MPN	RI	RH		
Antelope	22	18	01 (+)	02 (++)	05 (++)	01 (+)	-	-	7/9 (77.8)	11 /13 (84.6)
Greater cane rat	54	41	-	04 (+++)	02 (+)	01 (+)	-	01 (++)	7/8 (87.5)	34/46 (73.9)
Hare	11	7	-	-	-	-	-	01 (+)	0/1 (-)	07/10 (70.0)
African giant rat	5	4	-	02 (++)	01 (+)	-	-	-	2/3 (66.7)	02/02 (100)
Tree hyrax	3	2	-	-	-	-	01 (++)	-	0/1 (-)	02/02 (100)
Bush buck	1	1	-	-	01 (+)	-	-	-	1/1 (100)	-
Monitor lizard	2	2	01 (+)	-	-	-	-	-	1/1 (100)	01/01 (100)
Partridge	1	1	-	-	-	-	-	-	-	01/01 (100)
Python	3	3	-	-	-	-	-	-	-	03/03 (100)
Civet cat	3	3	-	-	-	-	-	-	-	03/03 (100)

**Total**	**102**	**82**	**2**	**8**	**9**	**2**	**1**	**2**	**18/24 (75)**	**64/81 (79.0)**

+ve, number positive; CH, cortical haemorrhages; PRCS, pitted and rough cortical surface; RN, renal nephrosis; MPN, multifocal pale nodules; RI, renal infarct; RH, renal hypoplasia; CP/PL, culturally positive /presence of lesions; CP/AL, culturally positive/absence of lesions, -, absent, +, mild, ++, moderate, +++, marked or severe.

**FIGURE 2:**
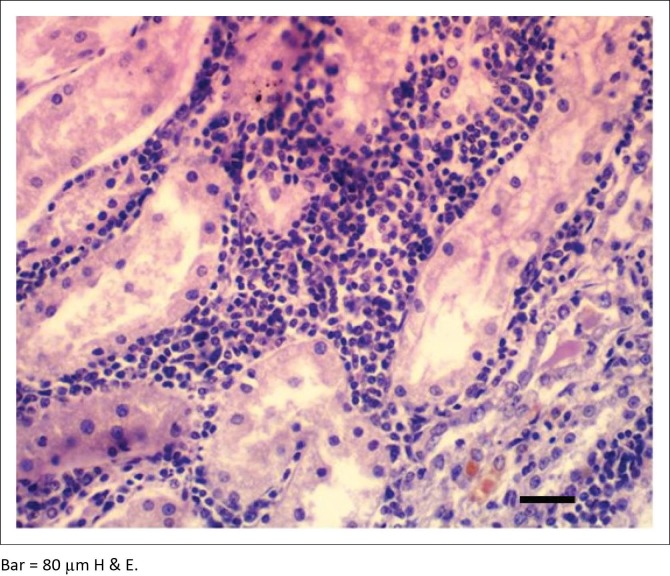
Kidney section showing tubular degeneration and necrosis, tubular atrophy with moderate diffuse interstitial lymphoplasmacytic cellular infiltration in antelope positive with *Leptospira* infection.

Results of histopathological changes, Warthin–Starry silver impregnation and IH of all the kidney sections collected are shown in the Tables 5 and 6. In all cases (both *Leptospira* positive and negative tissues), there were histopathological alterations in the kidneys. Tubular nephrosis (81.0%), IN (41.0%), interstitial fibrosis (20.0%) and protein cast (25.7%) were the most prominent histopathological changes observed in the kidney samples of both infected and non-infected animals, but these lesions were more marked in the infected animals. IN was mostly cortical. The IN was characterised by peritubular, perivascular and periglomerular lymphoplasmacytic inflammatory foci, and these coalesced in some sections (Figure 2). The severity of IN was mild in 35 (81.4%) animals, moderate in 7 (16.3%) and marked in only 1 (2.3%). GCR showed the highest prevalence of IN with 24 (55.8%) of the 43 wild animals that demonstrated the lesion.

**TABLE 5:** Renal localisation and tubular colonisation of *Leptospira* organism in wildlife using Warthin–Starry silver stain.

Species	Total (*n* = 93)	Number +ve (%)	Number −ve (%)	Tubular colonisation			Zonal localisation			Severity of tubular colonisation		
		
				PCT	DCT	COL	Cort.	CMJun.	Med.	+	++	+++
Antelope	19	7	12	6	4	2	3	3	1	7	-	-
Greater cane rat	51	42	9	27	20	13	15	22	5	25	14	3
Hare	10	7	3	6	4	1	2	4	1	5	2	-
African giant rat	3	3	-	1	1	1	1	1	1	3	-	-
Tree hyrax	3	-	3	-	-	-	-	-	-	-	-	-
Bush buck	1	-	1	-	-	-	-	-	-	-	-	-
Monitor lizard	2	2	-	2	-	1	2	-	1	2	-	-
Partridge	1	-	1	-	-	-	-	-	-	-	-	-
Python	3	2	1	1	1	-	-	1	1	1	1	-

**Total**	**93**	**63 (67.7%)**	**30 (32.3%)**	**43**	**30**	**18**	**23**	**31**	**10**	**43**	**17**	**3**

ve, positive; -ve, negative; PCT, proximal convoluted tubules; DCT, distal convoluted tubules; COL, collecting ducts; Cort., cortex; CMJun., corticomedullary junction; Med., medulla; -, absent; +, mild; ++, moderate; +++, marked or severe.

**TABLE 6:** Summary of the positive samples, histopathological changes, serovars identified and the methodologies used.

Number	Species	Total	Number +ve	Histopathological changes				Severity of IN			Serovars identified	Methodologies
	
				IN	TN	IF	PC	+	++	+++		
1	Antelope	22	18	10	20	4	10	8	2	-	*Leptospira grippotyphosa*	CI, MAT, WSss and IH
2	Greater cane rat	54	27	24	47	16	12	20	3	1	*Leptospira hardjo, Leptospira canicola* *Leptospira bratislava, Leptospira icterohaemorrhagiae* *Leptospira grippotyphosa*	CI, MAT, WSss and IH	
3	Hare	11	7	6	7	-	4	5	2	-	*Leptospira icterohaemorrhagiae, Leptospira canicola* *Leptospira bratislava*	CI and IH	
4	African giant rat	5	4	1	2	-	-	1	-	-	*Leptospira bratislava, Leptospira grippotyphosa*	CI, MAT and IH
5	Tree hyrax	3	2	-	3	-	-	-	-	-	ND	CI
6	Bush buck	1	1	-	1	-	-	-	-	-	ND	CI
7	Monitor lizard	2	2	1	2	1	1	1	-	-	ND	CI
8	Partridge	1	1	-	1	-	-	-	-	-	*Leptospira icterohaemorrhagiae*	CI, MAT
9	Python	3	3	1	2	-	-	1	-	-	*Leptospira grippotyphosa.*	CI, MAT
10	Civet cat	3	3	-	-	-	-	-	-	-	ND	CI

**Total**	**-**	**105**	**82**	**43**	**85**	**21**	**27**	**35**	**7**	**1**	**-**	**-**

+ve, positive; IN, interstitial nephritis; TN, tubular nephrosis; IF, interstitial fibrosis; PC, protein cast; -, absent; +, mild; ++, moderate; +++, marked or severe; ND, not determined; CI, cultural isolation; MAT, microscopic agglutination test; WSss, Warthin–Starry silver stain; IH, immunohistochemistry.

**FIGURE 3:**
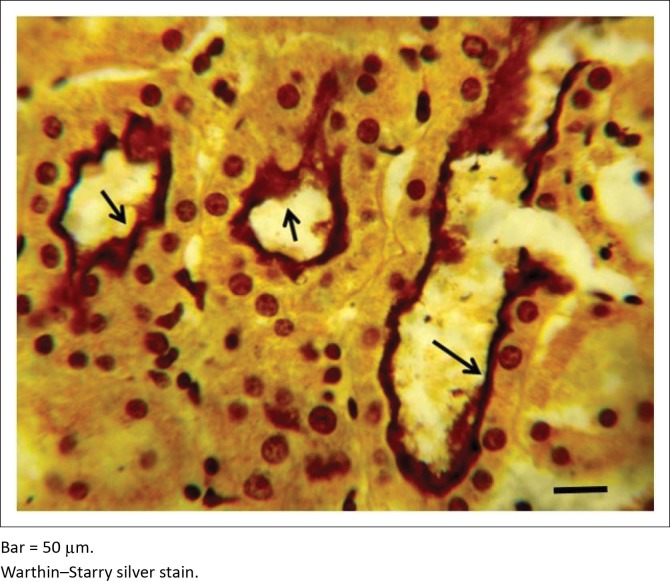
Photomicrograph of severely colonised proximal convoluted tubules and absence of interstitial mononuclear cells infiltration in the interstitium.

Of the 93 samples examined using WSss, 63 (67.7%) showed the presence of the organism. The morphological appearance of the organism was either intact or granular and in some cases intertwined, having a light brown to black appearance (Figure 3). Tubular colonisation showed that proximal convoluted tubules (PCT) demonstrated the highest prevalence compared with distal convoluted tubules (DCT) and collecting tubules. Despite the fact that PCT demonstrated the highest tubular colonisation, the corticomedullary junction showed the highest zonal localisation compared with the cortical and medullary zones. The severity of the tubular colonisation showed that the infection was mild in 43 (68.0%) kidneys, while it was moderate in 17 (27.0%) samples and severe in only 3 (5.0%) kidney samples.

**FIGURE 4:**
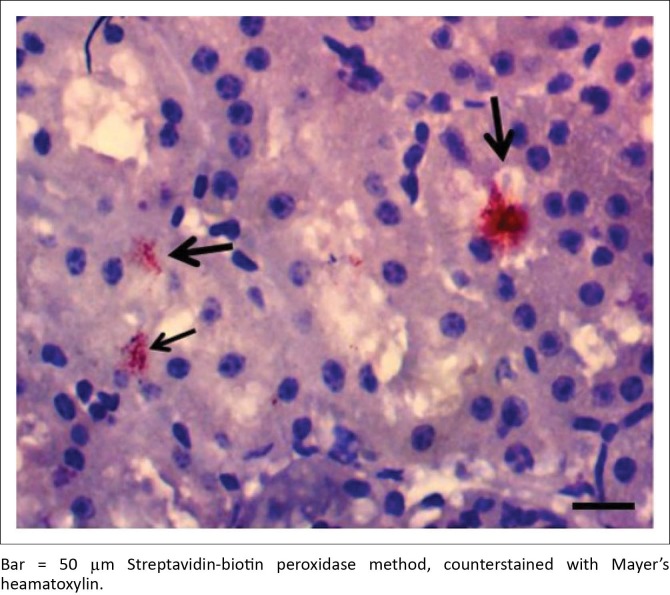
Kidney section of antelope showing immunoreactivity of *Leptospira interrogans* serovar hardjo antigen in the tubular lumen (arrows).

**FIGURE 5:**
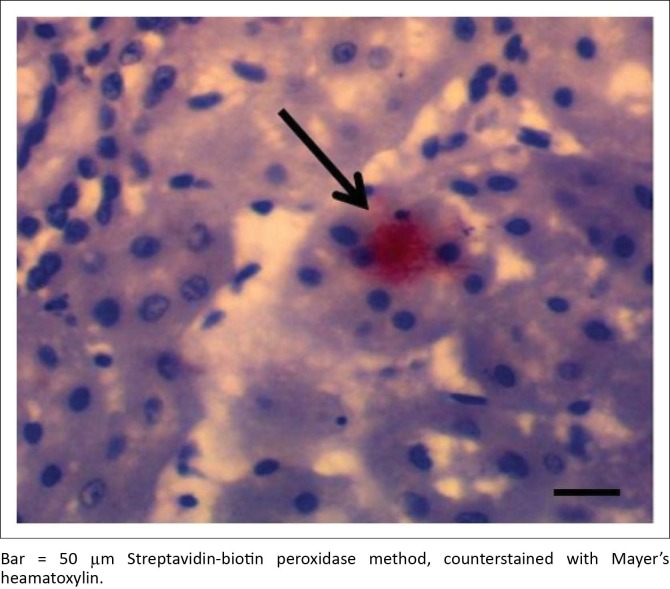
Kidney section of Hare showing strong immunoreactivity to *Leptospira interrogans* serovar bratislava antigen in the lumen of distal convoluted tubule (arrow).

IH of *Leptospira* antigens in the three kidney samples of hare were all positive with three different serovars detected (*L. icterohaemorrhagiae, L. canicola, L. bratislava*) (Figure 4). Two of the three samples in antelope were positive for *L. hardjo* (Figure 5), while *L. bratislava* and *L. gripptotyphosa* were detected in AGR. Only one of the three samples was positive in GCR for *L. gripptotyphosa.*

In summary, five different *Leptospira* serovars were isolated, characterised and detected from GCR using CI, MAT and IH. Three serovars each from antelope (*L. hardjo, L. pomona* and *L. gripptotyphosa*) and hare (*L. canicola, L. icterohaemorrhagiae, L. bratislava)* were detected, while two were detected from AGR and one each from python and partridge using either MAT or IH and both in some instances (Table 6). The isolates from tree hyrax, monitor lizard and civet cat were not characterised because of contamination of their samples.

## Discussion

This study revealed a high prevalence of leptospirosis in wild animals in the study area (78.1%) compared with other studies elsewhere (Millan et al. 2009; Shearer et al. 2014). It also showed the endemicity of the infection in wild animals in Abeokuta, Nigeria, and possibly in the southwest zone of the country. Factors responsible for this high prevalence are unknown, but it is possible to speculate that climatic conditions such as extended periods of rainfall and flooding, elevated temperatures throughout the year and high humidity favoured the prevalence of *Leptospira* in the study area.

During the last decade in Nigeria, undocumented cases of canine leptospirosis in dogs have been observed in various clinics and post-mortem examinations. Recently, serovars such as icterohaemorrhagiae, pomona, canicola, bratislava and gripptotyphosa have been serologically detected in dogs by Okewole and Ayoola, (2009) in Southwest Nigeria. In this study, these serovars were isolated and identified and possibly showed that wildlife might have been a source of infection to domestic animals.

There seemed to be no significant difference in the prevalence of infection with respect to sex in this study. This agrees with the studies of Hathaway, Blackmore and Marshall (1981) who observed no significant difference between male and female wildlife in their study.

Age-related occurrence of leptospirosis in the animals under investigation appears to be significant. The medium and large animals showed more prevalence than the small size. This might have been because of the fact that as the animal advances in age, the more the chances of exposure to the *Leptospira* infection. This is in agreement with the works of Hathaway et al. (1981) who observed marked differences in the age-specific prevalence of infection in adult animals in the wild.

Serology has been extensively used in the diagnosis of leptospirosis in the wild (Khan et al. 1991). However, there have been few studies on isolation, characterisation and pathology (Durfee et al. 1977). In this study, isolation and characterisation of *Leptospira* isolates were performed to ascertain the prevalent serovars. Of the 28 isolates for MAT, 16 (57%) isolates were characterised, while 12 (43%) were not. Serovars such as gripptotyphosa (*n* = 5), hardjo (*n* = 4), icterohaemorrhagiae (*n* = 3), pomona (*n* = 2), bratislava (*n* = 1) and canicola (*n* = 1) were identified in this study. This is in agreement with the earlier study of Okewole and Ayoola (2009) in which these serovars were identified serologically. The 12 unidentified isolates show that there are other serovars in wildlife in Nigeria apart from those recognised. Molecular characterisation of these isolates is underway and might likely show other unknown serotypes that were not previously documented.

The higher number of GCR in this study is in agreement with the works of Okarfor et al. (2013) who affirmed that GCR constitutes about 40.0% of mammalian species in the study area and their hunting rate is higher during the dry season compared with the rainy season. Of the 16 characterised isolates, GCR had the highest prevalence (31.3%) of isolates with five different serovars. The source and high rate of infection in GCR might be attributed to the nature of their ecosystem, since they live in an environment that enhanced the epidemiology of the *Leptospira* organism, such as marshy areas, river and lake banks (Adeyeye, Olaofe & Ogunjana 2012; Oboegbulem & Okoronkwo 1990). Moreover, GCR might possibly be a potential source of infection to other wild and domestic animals (especially cattle and dogs in peri-urban areas) and ultimately, a potential public health hazard to humans that consume smoked and inadequately cooked infected GCR.

Reports on isolation, characterisation and immunohistochemical detection of pathogenic leptospires in antelope are very rare in the literature, although serological evidence of *L. hardjo* has been demonstrated in 3.6% of 544 antelopes in Colorado, USA (Collins et al. 1981). In this report, of the 22 samples from antelope examined culturally and 6 isolates characterised with MAT, 18 (81.8%) and 4 (66.7%), respectively, were positive, while 7 (36.8%) and 2 (66.7%) were positive of 19 and 3 samples examined with WSss and IH, respectively. In this study, serovars hardjo, pomona and gripptotyphosa were the most prevalent serovars in this specie.

Studies on isolation and characterisation of pathogenic *Leptospira* from hare are also rare in the literature. In the work of Hathaway et al. (1981), five hares were examined serologically and culturally, but none of them were positive. In this study, 11 kidneys of hares were examined culturally and 7 (71%) were positive and 3 different serovars were detected immunohistochemically.

Although the numbers of civet cat, pythons, partridge, monitor lizard and bushbuck examined in this study were few, the 100% positivity shows that larger population of these animals might have been infected with leptospirosis. This might pose a serious concern for the conservation of these animal species in the wild.

The gross and histopathological changes observed in this study were consistent with those observed in domestic animals with renal *Leptospira* infection (Prescott et al. 2002). Previous studies have associated IN with leptospirosis in the literature (Rossetti et al. 2004; Scanziani, Sironi & Mandelli 1989; Sterling & Thiermann 1981; Yener & Keles 2001). In this study, of the 105 tissues examined histopathologically, IN was observed in 41.0% and the likely aetiology (leptospires) was present in 78.0% with CI and 67.7% using WSss. This possibly showed a positive correlation between IN and the presence of the organism in the renal tissues. The absence of IN in the kidney tissues that were positive to *Leptospira* organism might be because of the fact that the animals had overcome the infection and the inflammatory response had faded away with time, or that the little portion of the kidney examined might be devoid of such lesions as suggested by Ross et al. (2011). Interstitial fibrosis might have been because of previous subsiding inflammatory lesions induced by *Leptospira* organism and subsequent scarification.

On tubular colonisation, this study is in agreement with the report by Twigg and Cox (1976) in which PCT, DCT and collecting duct were consecutively colonised, but the number of animals involved in their study and the zonal localisation of *Leptospira* colonies in the renal parenchyma were not documented.

The use of diagnostic methods such as characterisation of leptospiral isolates using mAbs, WSss and IH is rare in the literature (Wild et al. 2002). To the best of our knowledge, the use of CI, characterisation, WSss, and IH have not been used to diagnose leptospirosis in wild animals in Africa and very few studies have been reported in other parts of the world (Cameron et al. 2008).

The high prevalence of leptospirosis (78.1%) in this study showed the endemicity of the infection in the wild. This prevalence might increase in the nearest future because of genetic mutation of the present serovars and possibly as a result of change in climatic conditions such as increase in daily temperature and longer period of rainy season. Thus, contact of wildlife with domestic animals should be prevented, especially in GCR domestication. Vaccination of domestic animals should be encouraged as part of preventive measures with a polyvalent vaccine that contain all identified serovars to provide full coverage. Future studies on the molecular characterisation of all the isolates in this study are warranted. This might detect and elucidate previously unrecognised pathogenic *Leptospira* organisms as well as the unidentified isolates in this study.

In conclusion, the high prevalence of leptospirosis in this study showed the endemicity of the disease in the wild and might be a potential source of infection to domestic animals and humans. Both gross and histopathological changes showed that wild animals are susceptible to leptospirosis and might be a potential source of infection to both domestic animals and humans. The study also illustrates the necessity of combining of different diagnostic methods in the confirmation of *Leptospira* infection. Serovars gripptotyphosa and hardjo had the highest prevalence in GCR and antelope, respectively, while serovars bratislava, canicola, icterohaemorrhagiae, pomona and gripptotyphosa were also detected using MAT and IH.
